# Microbial Phenolic Metabolites in Urine Are Inversely Linked to Certain Features of Metabolic Syndrome in Spanish Adolescents

**DOI:** 10.3390/antiox11112191

**Published:** 2022-11-05

**Authors:** Emily P. Laveriano-Santos, Paola Quifer-Rada, María Marhuenda-Muñoz, Camila Arancibia-Riveros, Anna Vallverdú-Queralt, Anna Tresserra-Rimbau, Ana María Ruiz-León, Rosa Casas, Ramon Estruch, Patricia Bodega, Mercedes de Miguel, Amaya de Cos-Gandoy, Jesús Martínez-Gómez, Gloria Santos-Beneit, Juan M. Fernández-Alvira, Rodrigo Fernández-Jiménez, Rosa M. Lamuela-Raventós

**Affiliations:** 1 Department of Nutrition, Food Science and Gastronomy, XIA, Faculty of Pharmacy and Food Sciences, Institute of Nutrition and Food Safety (INSA-UB), University of Barcelona, 08028 Barcelona, Spain; 2 Consorcio CIBER, M.P. Fisiopatología de la Obesidad y Nutrición (CIBERObn), Instituto de Salud Carlos III (ISCIII), 28029 Madrid, Spain; 3 LactApp Women Health, 08011 Barcelona, Spain; 4 Department of Endocrinology & Nutrition, CIBER of Diabetes and Associated Metabolic Diseases, Biomedical Research Institute Sant Pau, Hospital de la Santa Creu i Sant Pau, 08041 Barcelona, Spain; 5 Department of Internal Medicine, Hospital Clínic, Institut d’Investigacions Biomèdiques August Pi I Sunyer (IDIBAPS), University of Barcelona, 08036 Barcelona, Spain; 6 Mediterranean Diet Foundation, 08021 Barcelona, Spain; 7 Foundation for Science, Health and Education (SHE), 08008 Barcelona, Spain; 8 Centro Nacional de Investigaciones Cardiovasculares, 28029 Madrid, Spain; 9 The Zena and Michael A. Wiener Cardiovascular Institute, Icahn School of Medicine at Mount Sinai, New York, NY 10029, USA; 10 CIBER de Enfermedades Cardiovasculares (CIBERCV), 28029 Madrid, Spain; 11 Hospital Universitario Clínico San Carlos, 28040 Madrid, Spain

**Keywords:** microbiota, phytochemical, antioxidant compound, cardiovascular

## Abstract

(1) Background: To explore the association between microbial phenolic metabolites (MPM) and metabolic syndrome (MetS) and its clinical features in adolescents aged 12.02 ± 0.41 years. (2) Methods: a cross-sectional study was conducted in 560 participants at baseline in the SI! Program for Secondary Schools trial. The following MPM, coumaric acids (*m*-, *o*-, *p*-coumaric acids), dihydroxyphenylpropionic acid, dihydroresveratrol, enterolignans, gallic acid, hydroxybenzoic acids, hydroxyphenylacetic acid, hydroxytyrosol, protocatechuic acid, syringic acid, urolithins (A, B), and vanillic acid, were analyzed by HPLC-LTQ-Orbitrap-HRMS. MetS and its clinical features were defined in accordance with the International Diabetes Federation. (3) Results: Out of all MPM, urolithin A was inversely associated with the diastolic blood pressure z-score. Urolithin B was inversely associated with the MetS score and waist circumference z-score. Additionally, higher levels of gallic acid were associated with lower odds of presenting MetS (OR = 0.85, 95% CI: 0.77; 0.93) and abdominal obesity (OR = 0.93, 95% CI: 0.89; 0.98). Higher urolithin B levels were inversely associated with abdominal obesity (OR = 0.94, 95% CI: 0.89; 0.98) and high blood glucose (OR = 0.92, 95% CI:0.88; 0.96); (4) Conclusions: gallic acid, urolithin A and B were associated with lower odds of presenting MetS or some of its clinical features in adolescents. This is the first study that evaluates several MPM with MetS in adolescents, highlighting the importance of MPM on cardiometabolic health at early life stages.

## 1. Introduction

Metabolic syndrome (MetS) refers to a cluster of physiological, clinical features, biochemical, and metabolic conditions, including abdominal obesity, elevated blood pressure, dyslipidemia, and hyperglycemia, which are cardiovascular risk factors and are associated with insulin resistance [1,2]. MetS in childhood and adolescence is strongly associated with a high risk of maintaining MetS in adulthood and developing atherosclerosis and type 2 diabetes mellitus later in life [3]. In 2020, it was estimated that about 5% of adolescents worldwide had MetS; its prevalence was found to be unrelated to the wealth of individual countries [4] but linked to an increase in the rate of obesity stemming from an unhealthy diet and sedentary lifestyle [5]. Moreover, exposition to endocrine-disrupting chemicals, such as bisphenol A, parabens, and phthalates at early life stages contributes to obesity and other MetS features in adolescents [6,7].

As mentioned, diet is one of the modifiable factors strongly associated with obesity and MetS. The Mediterranean diet, characterized by foods rich in phenolic compounds, is known to reduce cardiometabolic risk factors [8,9,10]. The impact of dietary (poly)phenols on cardiometabolic health can be partly explained by their potential antioxidant action in preventing reactive oxygen species (ROS) production and cellular oxidative stress. The particular chemical structure of (poly)phenols makes them good electron or hydrogen atom donors, neutralizing ROS [11]. Prebiotic properties and the ability to modulate gut microbiota activity also have been attributable to phenolic compounds [12]. After ingestion, 85–90% of dietary (poly)phenols reach the large intestine, where they are metabolized by the gut microbiota into new compounds (metabolites) with potentially beneficial health effects [13,14]. The health effects of dietary (poly)phenols depend on the quantity consumed and their absorption, distribution, metabolism, and elimination in biological fluids [13]. The rate and extent of their absorption depend on the phenolic structure; whereas aglycones and some glycosides can be absorbed in the small intestine, esters and polymers are partly metabolized by gut microbiota and transformed into lower-molecular-weight compounds. These can be absorbed in the large intestine and reach the liver, where they may undergo further phase II metabolism. Once in the systematic circulation, phenolic metabolites are distributed to different target tissues and excreted through biological fluids, including urine, and can be identified through metabolomic approaches [13,15,16]. As dietary (poly)phenols undergo extensive modification, the forms that appear in human urine (mainly glucuronides and sulfates) are usually different from the parent compounds ingested in foods [13,14]. Several studies have documented the effect of phenolic metabolites on cardiometabolic outcomes, especially those derived from gut microbiota such as urolithins, phenolic acids (hydroxycinnamic acids, hydroxybenzoic acids, hydroxyphenylpropionic acids, hydroxyphenylacetic acids), enterolignans, and stilbenes (dihydroresveratrol) [13,17,18]. However, most of them have performed in adult populations.

Despite the plausible role of microbial phenolic metabolites (MPM) in cardiometabolic health and MetS [17,18] and the importance of investigating their role in young population, the aim of the present study was to evaluate the association between MPM and MetS and their clinical features in young adolescents in Spain using a targeted metabolomic approach.

## 2. Materials and Methods

### 2.1. Study Population

This is a sub-study nested within the SI! (Salud Integral-Comprehensive Health) Program for Secondary Schools trial (NCT03504059), a cluster-randomized controlled intervention trial that evaluated the effectiveness of different educational intervention strategies on cardiovascular health parameters in 1326 adolescents from 24 public Secondary Schools in Spain. A detailed description of the original study design and recruitment procedures has been published by Fernandez-Jiménez et al. [19].

The 601 subjects initially included in the present cross-sectional study had available baseline data on MPM and were selected by simple random sampling [20].

### 2.2. Assessment of Cardiometabolic Parameters

Waist circumference (WC) was measured using non-elastic tape; to minimize errors, the measurement was repeated three times, and the results were averaged [19]. Age- and sex-specific WC z-scores were calculated according to the National Health and Nutrition Examination III Survey data [21].

Systolic and diastolic blood pressure (SBP, DBP) (mm Hg) readings were taken with the subject in a sitting position using a digital device (Omron M6, OMROM Healthcare Co., Kyoto, Japan) according to standardized procedures [19]. Two readings were taken at intervals of 3–5 min, and if they differed by at least 5 mm Hg for DBP and/or 10 mm Hg for SBP, a third reading was taken. For this analysis, the lowest SBP and DBP values were considered. The mean arterial pressure (MAP) was obtained using the lowest values of SBP and DBP and calculated using the following formula: by the [(SBP − DPB)/3] + DBP. DBP and SBP z-scores were estimated following the cut-offs specified by the High Blood Pressure Working Group of the National Blood Pressure Education Program for children and adolescents [22].

The levels of blood glucose (BG), triglycerides (TG), and high-density lipoprotein cholesterol (HDL-c) were determined using a portable whole-blood analyzer in capillary blood samples collected early in the morning and fasting [19]. All of the measurements were performed by trained staff under standardized conditions.

Adolescents (aged 10 to <16 years) were considered to have MetS if they had abdominal obesity (WC ≥ 90th percentile) and at least two other clinical features, as defined by the International Diabetes Foundation [1]: (1) SBP ≥ 130 mm Hg or DBP ≥ 85 mm Hg, (2) TG ≥ 150 mg/dL, (3) HDL-c ≤ 40 mg/dL, or (4) BG ≥ 110 mg/dL. Additionally, a continuous MetS score was calculated following the methodology of Shafiee et al. [23] as the sum of standardized residuals for WC, MAP, HDL-c, TG, and BG, regressed for age and sex [23]. The HDL-c was multiplied by −1 because a low value of this parameter is an unfavorable factor of cardiometabolic risk. Thus, lower continuous MetS scores indicate a better cardiometabolic profile [23].

### 2.3. Determination of Urinary Phenolic Metabolites

#### 2.3.1. Reagents and Standards

Gallic acid (3,4,5-trihydroxybenzoic acid), 3-hydroxytyrosol (4-(2-hydroxyethyl)benzene-1,2-diol), protocatechuic acid (3,4-dihydroxybenzoic acid), 4-hydroxybenzoic acid, 3,4-dihydroxyphenylpropionic acid, 3′-hydroxyphenylacetic acid (2-(3-hydroxyphenyl)acetic acid), *o*-coumaric acid ((*E*)-2-(3-hydroxyphenyl)acetic acid), *m*-coumaric acid ((*E*)-3-(3-hydroxyphenyl)prop-2-enoic acid), *p*-coumaric acid ((*E*)-3-(4-hydroxyphenyl)prop-2-enoic acid), enterodiol ((2*R*,3*R*)-2,3-bis[(3-hydroxyphenyl)methyl]butane-1,4-diol), urolithin-A (3,8-dihydroxybenzo[c]chromen-6-one), and urolithin-B (3-hydroxybenzo[c]chromen-6-one) were purchased from Sigma-Aldrich Chemical Co. (St. Louis, MO, USA). 3′-hydroxytyrosol-3′-glucuronide ((2*S*,3*S*,4*S*,5*R*,6*S*)-3,4,5-trihydroxy-6-[2-hydroxy-5-(2-hydroxyethyl)phenoxy]oxane-2-carboxylic acid), dihydroresveratrol (5-[2-(4-hydroxyphenyl)ethyl]benzene-1,3-diol), and (+)-cis,trans-abscisic acid D6 ((2*Z*,4*E*)-3-methyl-5-[(1*S*)-3,5,5-trideuterio-1-hydroxy-6,6-dimethyl-4-oxo-2-(trideuteriomethyl)cyclohex-2-en-1-yl]penta-2,4-dienoic acid) were obtained from Santa Cruz (Santa Cruz Biotechnology, Santa Cruz, CA, USA). 3-hydroxybenzoic acid, vanillic acid (4-hydroxy-3-methoxybenzoic acid), syringic acid (4-hydroxy-3,5-dimethoxybenzoic acid), enterolactone ((3*R*,4*R*)-3,4-bis[(3-hydroxyphenyl)methyl]oxolan-2-one), and creatinine were obtained from Fluka (St. Louis, MO, USA). The standards were stored, protected from light, and in powder form. Methanol and acetonitrile grade were obtained from Sigma-Aldrich Chemical Co. (St. Louis, MO, USA), formic acid (≥98%) from Panreac Química S.A. (Barcelona, Spain), and ultrapure water (Milli-Q) from a Milli-Q system (Bedford, MA, USA).

#### 2.3.2. Urine Sample Collection and Treatment for MPM Analysis

Baseline spot urine samples were collected in the morning and fasting in 1 mL polyethylene containers and stored at −80 °C prior to analysis. The extraction of phenolic compounds was carried out using a method developed by our research group [20]. For the sample preparation, urine (1 mL) was acidified with 2 μL of formic acid and centrifuged (4 min, 15,000× *g*, 4 °C) one time. The supernatant was then loaded onto Waters Oasis 96-well reversed-phase phase extraction plates (30 µm) (MA, USA) prior to plate activation with 1 mL of methanol and 1 mL of 1.5 M formic acid. Sample clean-up was performed with 500 μL of 1.5 M formic acid and 0.5% methanol, and elution was achieved using 1 mL of methanol (acidified with 0.1% formic acid). Then, the eluted fraction was evaporated to dryness under a nitrogen stream at room temperature and reconstituted with Milli-Q water (0.05% formic acid) up to 100 μL. The extract was filtered with a 0.22 µm polytetrafluoroethylene 96-well plate filter from Millipore (Burlington, MA, USA). (+)-cis,trans-abscisic acid d6 (500 μg/L) was used as the internal standard.

The calibration curves were prepared following the same procedure by spiking synthetic urine at nine different concentrations of standard mixtures according to our previous method [20].

#### 2.3.3. Chromatographic Conditions

MPM were identified and quantified by reverse-phase high-performance liquid chromatography coupled to linear trap quadrupole Orbitrap high-resolution mass spectrometry (HPLC -LTQ-Orbitrap-HRMS), as previously described [20]. The liquid chromatography system was an Accela chromatograph (Thermo Scientific, Hemel Hempstead, UK) equipped with a quaternary pump and a thermostated autosampler set at 4 °C. The HPLC column employed was a Kinetex F5 100 Å (50 × 4.6 mm i.d., 2.6 µm particle size from Phenomenex (Torrance, CA, USA) at 40 °C. The mobile phases consisted of water/0.05% formic acid (A) and acetonitrile/0.05% formic acid (B), with a flow rate of 0.5 mL min^−1^. The non-linear gradient program started with 2% of solvent B, reaching 8% solvent B at 2.5 min, 20% solvent B at 7 min, 30% solvent B at 9 min, 50% solvent B at 11 min, 70% solvent B at 12 min, and 100% of solvent B at 15 min. The initial conditions were re-established at 16.5 min and maintained up to 21.5 min.

For accurate mass measurements, the HPLC system was coupled with an LTQ-Orbitrap-HRMS (Thermo Scientific, Hemel Hempstead, UK) equipped with an electrospray ionization (ESI) source on the negative mode, with the following parameters: source voltage, 5 kV; sheath gas, 50 units; auxiliary gas, 20 units; sweep gas, 2 units; and capillary temperature, 375 °C. Mass spectra (MS) were acquired in profile mode with a setting of 30,000 resolution at m/z 400. The mass range in Fourier transform mass spectrometry mode was from *m*/*z* 100 to 2000, in combination with product ion scan experiments (resolution range 15,000–30,000 full-width at half maximum) [20].

MPM identification, described in detail by Laveriano-Santos et al., was based on their accurate mass measurements with an error of 5 ppm and isotopic patterns, as well as the existing literature [20]. Xcalibur 3.0 and Trace Finder version 4.1 (Thermo Fisher Scientific, San Jose, CA, USA) were applied for the instrument control and chromatographic data analysis.

In this study, 54 MPM were identified and quantified (aglycones and phase II metabolites in glucuronide and sulfate form) (Table S1). MPM values below the limit of quantitation (LOQ) were replaced by the mean LOQ/2.

#### 2.3.4. Creatinine Analysis

Urinary creatinine was measured using a Jaffé alkaline picrate method adapted for microtiter 96-well plates described by Medina-Remón et al. [24]. Briefly, 3 μL of a spot urine sample was mixed with 60 μL of aqueous picric acid solution (1%) and 5 μL of sodium hydroxide (10%) and maintained in darkness for 15 min. After adding 232 μL of Milli-Q water, the absorbance was measured at 500 nm by a UV–vis spectrophotometer; all of the experiments were performed in triplicate. The calibration curve was prepared with a creatinine standard to quantify the creatinine concentrations in the spot urine samples. MPM concentrations were normalized by creatinine and expressed as µg MPM/g creatinine.

### 2.4. Assessment of Covariates

The energy intake (kcal/day) was estimated from a semi-quantitative food frequency questionnaire [25] using Spanish food composition tables [26,27].

Physical activity was measured by accelerometry (Actigraph wGT3X-BT, ActiGraph, Pensacola, FL, USA). The participants wore accelerometers on the non-dominant wrist for 7 consecutive days [19]. Moderate-to-vigorous physical activity (min/day) was estimated according to Chandler et al. (2016) [28]. Physical activity self-reported information obtained by questionnaires was used if accelerometer data were missing (*n* = 13; 2%) [19,29]. Finally, for this study, physical activity was categorized based on the recommendation of the World Health Organization as (1) below or (2) at least 60 min/day of moderate-to-vigorous physical activity [30]. Sexual maturity status was established using pictograms according to the Tanner & Whitehouse method [31].

Information about household income was obtained from a validated questionnaire answered by the parents [19]. Household incomes were categorized as low, medium, or high based on salaries in Spain [32].

### 2.5. Statistical Analysis

Stata software version 16.0 (StataCorp., College Station, TX, USA) and R 4.2.1 (R Studio, 250 Northern Ave, Boston, MA, USA) were used to perform the data analyses. The data are expressed as mean ± SD and frequency (percentage).

MPM outlier values were identified using a robust mean absolute deviation method (MAD) [33], and 41 participants (7% of the selected sample) with MPM values of more than 3MAD were excluded from the analysis, leaving a total of 560 participants (Figure S1). The MPM were divided into 14 groups according to their chemical structure, and each group included all the aglycone, glucuronide, and sulfate forms. Thus, statistical analysis was carried out with the following MPM groups: coumaric acids (including *p*-, *o*-, and *m*-coumaric acid), dihydroxyphenylacetic acid, dihydroresveratrol, enterodiol, enterolactone, gallic acid, hydroxybenzoic acids (including 3- and 4-hydroxybenzoic acids), hydroxyphenylacetic acid, hydroxytyrosol, protocatechuic acid, syringic acid, urolithin A, urolithin B, and vanillic acid. As the MPM levels had a skewed distribution, the analysis was carried out with their natural log-transformed concentrations.

Association between the log-transformed MPM (continuous) and cardiometabolic health parameters (WC, DBP, SBP, BG, TG, HDL-c, and MetS scores, all continuous) was determined using mixed-effects linear regression analyses. The fixed effect included two different models: model 1 was adjusted for sex (females/males) and age (continuous, years), whereas model 2 was model 1 plus Tanner maturation stage (from I to V), physical activity (≥60 min/<60 min moderate-to-vigorous physical activity), household income (low/medium/high), and energy intake (continuous, kcal/day). Mixed-effects logistic regression was used to assess the associations between the log-transformed MPM (continuous) and MetS presence (yes/no), as well as its clinical features, ((1) WC ≥ 90th percentile, (2) SBP ≥ 130 mm Hg or DBP ≥ 85 mm Hg, (3) TG ≥ 150 mg/dL, (4) HDL-c ≤ 40 mg/dL, (5) BG ≥ 110 mg/dL), adjusting for the same covariates as mentioned above. Municipality and schools were considered as random effects in all adjusted models. The results are expressed as unstandardized β coefficients or odds ratios (OR) and their 95% confidence intervals (CI). To compensate for multiple testing, the Benjamin–Hochberg procedure was applied, considering a false discovery rate (FDR) < 0.05 as significant [34], although all *p* values below 0.05 are provided. To summarize the regression analyses, Forest plots were applied using the “forester” package for R software. An analysis flowchart was designed to visualize all the statistical analyses (Figure S2).

## 3. Results

### 3.1. Participant Characteristics

Out of a total sample of 601 adolescents, 560 (54% female) aged 12.0 ± 0.4 years were included in the study (Figure S1). The following mean measurements were obtained for this sub-study: WC 73.6 cm, SBP 105.2 mm Hg, DBP 62.5 mm Hg, BG 102.9 mg/dL, TG 87.9 mg/dL, and HDL-c 62.4 mg/dL. Overall, 72% of the participants reported at least 60 min/day of moderate-to-vigorous physical activity. Regarding sociodemographic factors, more than a third reported a high household income (Table 1).

Sixteen participants were diagnosed with MetS, and the overall crude prevalence of MetS was 3%. 240 (43%) participants had one or two components of MetS, while 304 (54%) had none. The mean MetS score was 0.01 ± 3.04. Regarding MetS clinical features, 122 (22%) participants presented abdominal obesity (WC ≥ 90th), 2 (less than 1%) had high blood pressure (≥130 mm Hg SBP or ≥85 mm Hg DBP), 43 (8%) had high TG (≥150 mg/dL), 32 (6%) had low HDL-c (≤40 mg/dL), and 159 (28%) had high BG (≥110 mg/dL) (Table 1).

### 3.2. Association between MPM and Cardiometabolic Health Parameters

**Figure 1:**
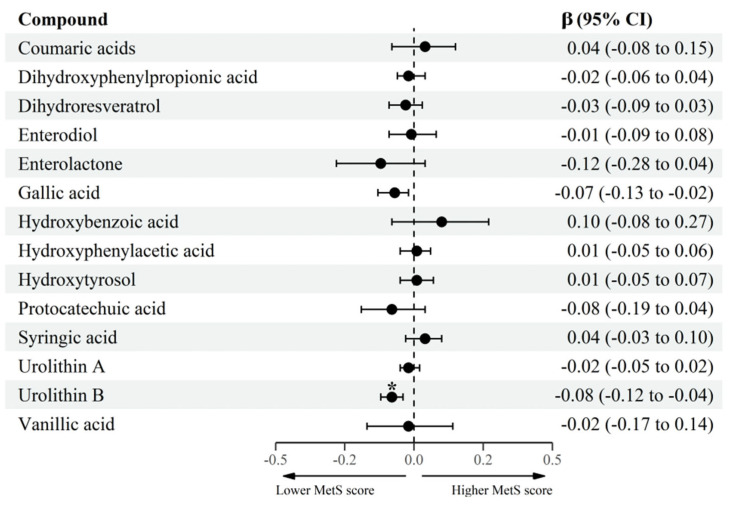
Association between log-transformed groups of MPM and MetS score in adolescents. β coefficient, CI confidence interval, MetS metabolic syndrome, MPM microbial phenolic metabolites. Mixed-effects linear regression between log-transformed MPM and MetS score. Fixed effect: sex (female/male), age (continuous, years), Tanner maturation stage (score from I to V), physical activity (≥60 min/<60 min moderate-to-vigorous physical activity), household income (low/medium/high), and energy intake (continuous, kcal/day). Municipality and schools were considered as random effects). * *p*-adjusted for multiple-testing using the Benjamin–Hochberg procedure considering a false discovery rate < 0.05 as significant.

**Figure 2:**
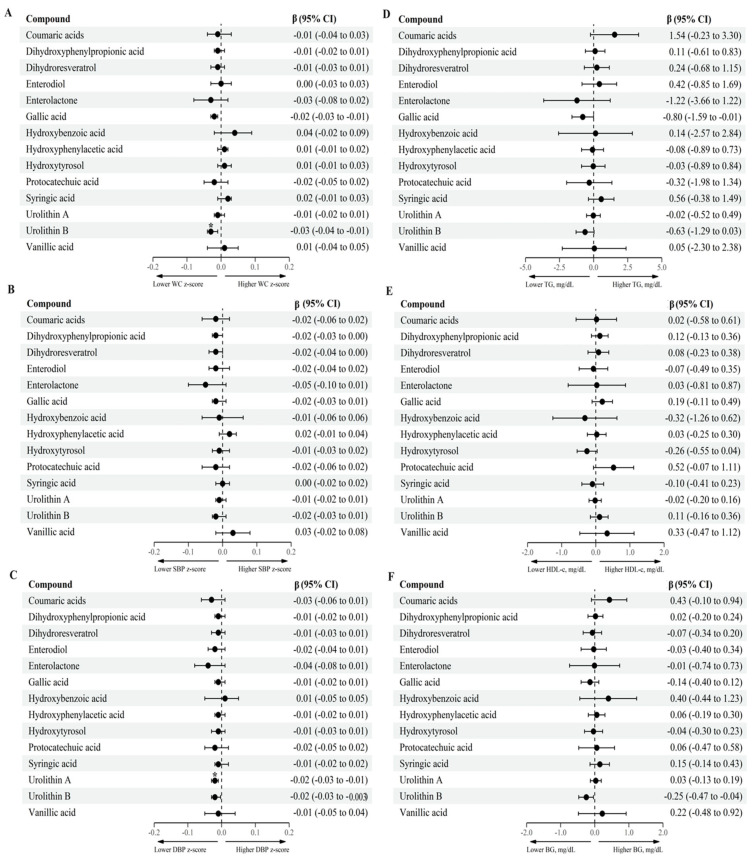
Association between log-transformed groups of MPM and cardiometabolic health parameters in adolescents. (**A**): WC z-score, (**B**): SBP- z-score, (**C**): DBP z-score, (**D**): TG, mg/dL, (**E**): HDL-c, mg/dL, (**F**): BG, mg/dL. β coefficient, BG blood glucose, CI confidence interval, DBP diastolic blood pressure, HDL-c high-density lipoprotein-cholesterol, MPM microbial phenolic metabolites, SBP systolic blood pressure, TG triglycerides, WC waist circumference. Mixed-effects linear regression between log-transformed MPM and cardiometabolic health parameters. Fixed effect: sex (female/male), age (continuous, year), Tanner maturation stage (score from I to V), physical activity (≥60 min/<60 min moderate-to-vigorous physical activity), household income (low/medium/high), and energy intake (continuous, kcal/day). Municipality and schools were considered random effects. * *p*-adjusted for multiple-testing using the Benjamin–Hochberg procedure, considering a false discovery rate < 0.05 as significant.

Mixed-effects linear regression models were generated to evaluate the association between the log-transformed groups of MPM and the cardiometabolic health parameters. In the fully adjusted multivariable model, adjusted for sex, age, Tanner maturation stage, physical activity, household income, and energy intake, after multitesting adjustment for FDR, the results showed that higher urolithin B levels (aglycone, glucuronide, and sulfate forms) were associated with lower MetS score (β= −0.08, 95% CI: −0.12; −0.04) (Figure 1 and Table S2), but also with lower WC z-scores (β= −0.03, 95% CI: −0.04; −0.01) (Figure 2 and Table S2). Higher concentrations of urolithin A (aglycone, glucuronide, and sulfate forms) were associated with lower DBP z-scores (β= −0.02, 95% CI: −0.03; −0.01) (Figure 2 and Table S2).

### 3.3. Association between MPM and MetS and Its Clinical Features

**Figure 3:**
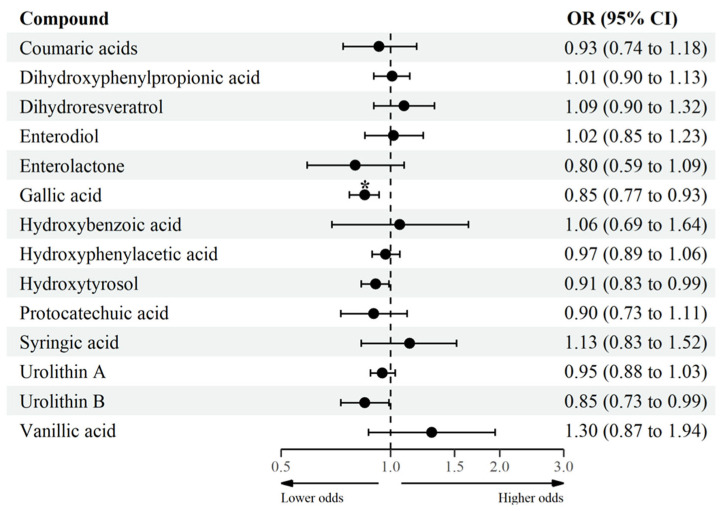
Log-transformed groups of MPM with MetS in adolescents. CI confidence interval, MetS metabolic syndrome; MPM microbial phenolic metabolites, OR odds ratio. Mixed effect logistic regression between groups of MPM (log-transformed, continuous) and MetS (dichotomous). Fixed effect: sex (female/male), age (continuous, years), Tanner maturation stage (score from I to V), physical activity (≥60 min/<60 min moderate-to-vigorous physical activity), household income (low/medium/high), and energy intake (continuous, kcal/day). Municipality and schools were considered as random effects. * *p*-adjusted for multiple-testing using the Benjamin–Hochberg procedure, considering a false discovery rate < 0.05 as significant.

**Figure 4:**
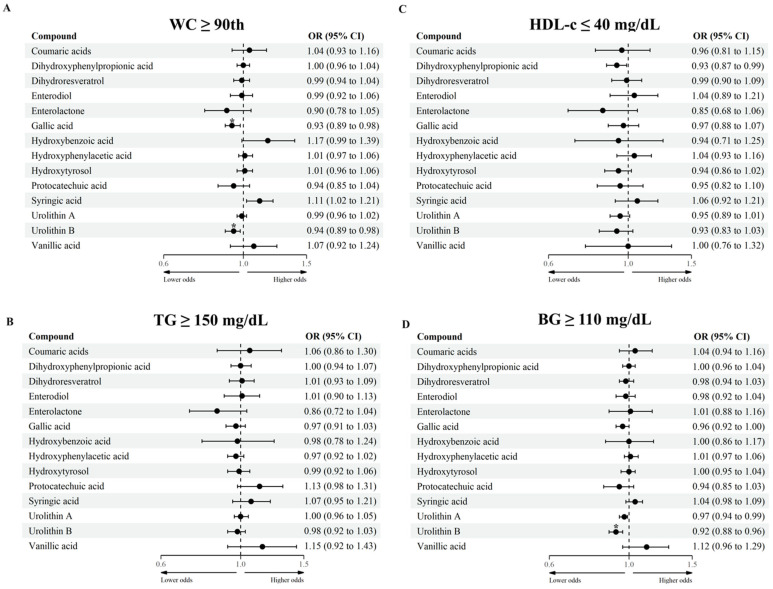
Log-transformed groups of MPM with MetS clinical features in adolescents. (**A**): WC ≥ 90th, (**B**): TG ≥ 150 mg/dL, (**C**): HDL-c ≤ 40 mg/dL, (**D**): BG ≥ 110 mg/dL. BG blood glucose, CI confidence interval, HDL-c high-density lipoprotein-cholesterol, MPM microbial phenolic metabolites, TG triglycerides, WC waist circumference. Mixed-effects logistic regression between groups of MPM (log-transformed, continuous) and MetS clinical features (dichotomous). Fixed effect: sex (female/male), age (continuous, years), Tanner maturation stage (score from I to V), physical activity (≥60 min/<60 min moderate-to-vigorous physical activity), household income (low/medium/high), and energy intake (continuous, kcal/day). Municipality and schools were considered random effects. High blood pressure was not considered in the statistical analysis as only 2 participants had this condition (systolic blood pressure ≥ 130 mm Hg or diastolic blood pressure ≥ 85 mm Hg), and therefore analysis did not converge. * *p*-adjusted for multiple-testing using the Benjamin–Hochberg procedure, considering a false discovery rate < 0.05 as significant.

The association between MPM and MetS and its clinical components was assessed through mixed-effects logistic regression analyses (Figure 3 and Figure 4 and Table S3). After adjustment for an FDR, higher gallic acid levels (aglycone, glucuronide, and sulfate forms) were associated with lower odds of presenting MetS (OR = 0.85, 95% CI: 0.77; 0.93) and abdominal obesity (OR = 0.93, 95% CI: 0.89; 0.98) defined as WC ≥ 90th. Similarly, participants with higher urolithin B levels (aglycone, glucuronide, and sulfate forms) had lower odds of having abdominal obesity (OR = 0.94, 95% CI: 0.89; 0.98) and high BG (OR = 0.92, 95% CI:0.88; 0.96). High blood pressure was not considered in the statistical analysis as only 2 participants (less than 1%) presented this condition (SBP ≥ 130 mm Hg or SBP ≥ 85 mm Hg), and therefore the analysis did not converge.

## 4. Discussion

The results of the present cross-sectional study of 560 adolescents show that higher urinary excretion of phenolic metabolites (gallic acid, and urolithin A and B) was associated with a lower WC z-score, DBP z-score, and MetS score and reduced odds of presenting MetS or some of its clinical features (abdominal obesity and high BG), after adjustment for sex, age, Tanner maturation stage, physical activity, household income, and energy intake.

The potential role of MPM on MetS and its clinical features have been documented in several studies; however, most of them have been conducted in adult, in vitro, or animal models.

We observed that higher levels of urinary gallic acid (aglycone, glucuronide, and sulfate forms) were associated with lower odds of having MetS and abdominal obesity. Gallic acid is a small phenolic acid, widespread in fruits and vegetables as a free molecule in aglycone or esterified form [14,35]. Moreover, gallic acid is one of the main microbial metabolites arising from hydrolysable tannins (gallotannins), anthocyanins, and galloylated catechin (epigallocatechin gallate and epicatechin gallate) [14]. According to Esteban-Torres et al., gut microbiota phyla such as *Firmicutes*, *Proteobacteria*, and *Actinobacteria* contain gallate decarboxylase and/or tannase enzymes responsible for the degradation of dietary gallates and tannins, transforming into simpler compounds, such as gallic acid and pyrogallol [36]. The therapeutic properties of gallic acid related to its strong antioxidant activities have been demonstrated in several studies [35], but its effects have been scarcely studied in adolescents. Gallic acid promotes mitochondrial biogenesis, a vital process for maintaining cellular homeostasis in terms of energy production and heat generation, which are closely associated with obesity, diabetes, and other metabolic disorders [35]. In animal models, gallic acid and epicatechin gallate induce thermogenesis and mitochondrial biogenesis in brown adipocytes [37]. Gallic acid and its ester derivatives improve the function of several mitochondrial enzymes involved in cellular energy homeostasis regulating body weight and lipid and glucose metabolism [35,38]. The thermogenic effect of galloylated catechins, especially epigallocatechin gallate, on weight loss has been studied in human adults. The results from a meta-analysis based on five randomized, double-blind, placebo-controlled clinical trials involving 112 participants showed that a moderate daily intake of epigallocatechin gallate (300–600 mg dose) for a period of 2 days to 12 weeks increases energy expenditure in 158 kJ/day or 38 kcal/day compared to placebo [39]. In the same study, epigallocatechin gallate supplementation reduced WC and body fat mass but not body mass index [39]. Another clinical trial showed that daily supplementation of green tea extract capsules, with a high concentration of galloylated catechins (approximately 1000 mg) for twelve weeks, reduced body weight and increased energy expenditure and fat oxidation in sixty subjects aged 40–60 years and with overweight [40].

Concerning glucose metabolism, which is closely linked to MetS, gallic acid can reduce BG levels by suppressing glucose absorption in the intestinal cell via the inhibition of sodium-dependent transporters [41]. Additionally, gallic acid stimulates glucose transporter 4 translocation and increases glucose uptake activity in adipocytes, thus demonstrating its antihyperglycemic properties [13,42,43,44]. A study of streptozotocin-induced diabetic rats reported that supplementation of 10 and 20 mg/kg body weight of gallic acid for 21 days decreased body weight, glycosylated hemoglobin, glucose-6-phosphatase activity, pancreatic lipid peroxidation, and pancreatic damage [45]. In a randomized clinical trial conducted in thirty-seven individuals aged 16–65 years with MetS, the daily consumption of a beverage (650 mL) with high gallic acid content for twelve weeks did not show changes in BG in the placebo and intervention group [46]. However, a significant decrease in inflammatory biomarkers (interleukin 6 and 8-isoprostane), closely associated with MetS, was observed in the intervention group [46]. These finding showed in the literature agree with our results and provide strong support for the possible effect of gallic acid on MetS and obesity.

In the present study, higher levels of urinary urolithin A and B (aglycone, glucuronide, and sulfate forms) were associated with better cardiometabolic health parameters (lower MetS score, WC z-score, and DBP z-score) and reduced odds of presenting abdominal obesity and high BG (the latter only with urolithin B). Urolithins are the final metabolites of ellagitannins, the main group of hydrolysable tannins [13,14,47], which are found in nuts and red fruits, including grapes, pomegranates, and some berries. Ellagitannins are broken down into ellagic acids, which are transformed into urolithins by the action of the gut microbiota [14,47,48]. Urolithins are metabolized in enterocytes and hepatocytes into glucuronide, sulfate, and methylated phase II metabolites and are excreted in breast milk and urine [13,49]. Several studies in vitro and in animal models and some human clinical trials have revealed a protective effect of urolithins (ellagitannin metabolites) against obesity, type 2 diabetes mellitus, cardiovascular diseases, and some cancers [17,18,47]. Regarding blood pressure, we found that a higher excretion of urolithin A was related to a lower DBP score, in agreement with Istas G. et al., who reported a beneficial effect of plasma urolithins on endothelial function in healthy individuals aged 18–35 years after the consumption of 200 and 400 g of raspberries. Additionally, a reduction in blood pressure (central and peripheral) was observed after raspberry intake [50]. The results from a meta-analysis conducted in thirty randomized clinical trials showed that pomegranate and nut (sources of ellagitannins) supplementation did not have a significant reduction in SBP and DBP levels in adults. However, in a separate analysis, differences in the regulation of blood pressure were observed; meanwhile, pomegranate intake significantly reduced DBP levels, and nut intake increased DBP [51]. The association between urolithin A and blood pressure found in our study could be explained by the antioxidant and anti-inflammatory properties of Urolithin A by decreasing inflammation markers (prostaglandins E2, cyclo-oxigenase-2, and microsomal prostaglandins E synthase-1) and inhibiting the activation of mitogen-activated protein kinase and nuclear factor kappa B [52]. Moreover, urolithin A mediates vascular function by inhibiting the adhesion of monocytes and endothelial cell migration, as well as activating endothelial nitric oxide synthase and releasing nitric oxide [53,54]. We also observed that higher urinary levels of urolithin B were associated with lower odds of presenting abdominal obesity, which could be attributed to its enhancing effects on thermogenesis, lipolysis, fatty oxidation, and inhibition of lipogenesis and adipogenesis [47]. Significant interindividual variation has been observed in urolithin production and excretion, with an effect on cardiometabolic health [14,17,18,48]. Based on urinary excretion, three urolithin metabotypes (UMs) have been identified: UM-A individuals produce only urolithin A, those with UM-B produce urolithin B and isourolithin A, and those with UM-0 are nonproducers [14,17,18]. UMs could be used as biomarkers of gut microbiota [55]. In a Spanish cohort of 415 children and adolescents aged 5–17 years, who consumed 25 g of walnuts (peeled raw) or 250 mL of pomegranate juice daily for three days, UM-B was associated with higher odds of overweight-obesity defined by body mass index cut-offs compared to UM-A. In this study, urolithin B was not evaluated separately; therefore, our results cannot be comparable. In our study, we did not evaluate urolithin metabotypes, and therefore their possible associations with cardiometabolic parameters cannot be determined [55]. Additionally, we found that urolithin B was associated with lower odds of presenting high BG. By contrast, Selma et al. did not find a relationship between urolithin B + isourolithin A in urine and BG in fifty MetS subjects aged 42–61 years [17]. Our findings could be explained by the protective effect of urolithins on the exocrine pancreas through the inhibition of inflammatory signaling pathways, autophagy activation, and maintenance of mitochondrial function in pancreatic cells [48]. Hyperglycemia plays a pivotal role in the pathogenesis of cardiometabolic diseases through the stimulation of cellular oxidative stress, mitochondrial dysfunction, and inflammation [56]. According to the results from an in vitro study, 1 µM urolithin B and B-glucuronide prevents inflammatory responses in rat cardiac myocytes exposed to high glucose concentrations. Thus, the authors highlighted the importance of a regular intake of ellagitannin-rich foods in the modulation of pro-inflammatory mediators in hyperglycemic conditions [11,57]

In our cross-sectional study, coumaric acids (including *p*-, *o*-, and *m*-coumaric acid), dihydroxyphenylacetic acid, dihydroresveratrol, enterodiol, enterolactone, hydroxybenzoic acids (including 3- and 4-hydroxybenzoic acids), hydroxyphenylacetic acid, hydroxytyrosol, protocatechuic acid, syringic acid, and vanillic acid; were not associated with MetS and its clinical features in adolescents after the full-adjustment for confounder variables, in contrast with the literature reported in studies conducted in human adults, in vitro, and in animal models [13,43,58,59].

To our knowledge, this is the first observational study to investigate the associations of several MPM with cardiometabolic parameters and MetS and its clinical components in young adolescents. Another strong point of the study is the precise extraction of phenolic metabolites from urine samples and the use of a novel targeted metabolomic approach for their highly accurate identification and quantification. However, several limitations also need to be acknowledged. First, as this is a cross-sectional study, causal associations cannot be assumed, and the possibility of reverse causation cannot be excluded. Second, the scope of the study did not include the elucidation of molecular mechanisms underlying these associations. Third, inter-individual variation could affect the bioavailability of MPM such as food matrix and processing, gut microbiota profile, genetic polymorphisms, enzymatic capacity (especially phase II enzymes) of the host [42,60], biological rhythms [61], and environmental exposure (e.g., phthalates and phenols as endocrine disruptors related to obesity indicators) [6,7], all of them were not determined in our study. Fourth, as the participants did not use accelerometers in water sports or during competitions, the physical activity levels may have been underestimated. Finally, as some participants were probably evaluated in non-fasting conditions, the BG might be overestimated: only 1/560 participants reported non-fasting, but BG levels seem too high for a healthy adolescent population.

## 5. Conclusions

In summary, a higher concentration of MPM (gallic acid and urolithin A and B) was associated with better cardiometabolic health (lower MetS scores) in a sub-sample of adolescents enrolled at baseline in the SI! Program for Secondary Schools trial. Additionally, MPM were associated with lower odds of presenting MetS or some of its clinical features, such as abdominal obesity and high BG. These findings highlight the importance of (poly)phenols from gut microbiota metabolism on cardiometabolic health at early life stages. Further prospective analysis and clinical trials are strongly warranted to investigate the effect of MPM on cardiometabolic health in this young population.

## Figures and Tables

**Table 1:** General characteristics of the participants (*n* = 560).

Characteristics	Total
Female, *n* (%)	304 (54)
Age, years	12.0 ± 0.4
WC, cm	73.6 ± 11.4
SBP, mm Hg	105.3 ± 11.2
DBP, mm Hg	62.5 ± 9.3
BG, mg/dL	102.9 ± 14.2
TG, mg/dL	87.9 ± 47.2
HDL-c, mg/dL	62.4 ± 16.5
MetS score	0.01 ± 3.04
MetS, *n* (%)	16 (3)
Clinical features	
Abdominal obesity, *n* (%)	122 (22)
High blood pressure, *n* (%)	2 (0.4)
Low HDL-c, *n* (%)	32 (6)
High TG, *n* (%)	43 (8)
High BG, *n* (%)	159 (28)
Total energy intake, kcal/day	2492.1 ± 586.4
Moderate-to-vigorous physical activity, *n* (%)	
<60 min/day	156 (28)
≥60 min/day	404 (72)
Household income, *n* (%)	
Low	173 (32)
Medium	144 (27)
High	216 (41)

Values are expressed as mean ± SD for continuous variables or as a frequency (percentage) for categorical variables. BG blood glucose, DBP diastolic blood pressure, HDL-c high-density lipoprotein-cholesterol, SBP systolic blood pressure, MetS metabolic syndrome, TG triglycerides, WC waist circumference.

## Data Availability

The database analyzed during this cross-sectional study is not publicly available. Requestors wishing to access the database used in this study can make a request to the Steering Committee chair of the SI! Program trial: gsantos@fundacionshe.org, rodrigo.fernandez@cnic.es, juanmiguel.fernandez@cnic.es, RESTRUCH@clinic.cat, lamuela@ub.edu, bibanez@cnic.es, vfuster@cnic.es.

## Supplementary Materials

The following supporting information can be downloaded at: https://www.mdpi.com/article/10.3390/antiox11112191/s1, Figure S1: Flow-chart of study participants; Figure S2: Analysis flow-chart; Table S1: Phenolic metabolites identified and quantified in urine by HPLC-LTQ-Orbitrap-HRMS; Table S2: Log-transformed group of MPM and cardiometabolic health parameters; Table S3: Log-transformed group of MPM and MetS and its components.

## Abbreviations

BG	blood glucose
CI	confidence interval
DBP	diastolic blood pressure
FDR	false discovery rate
HDL-c	high-density lipoprotein cholesterol
HPLC-LTQ-Orbitrap-HRMS	High-performance liquid chromatography coupled to a linear trap quadrupole Orbitrap high-resolution mass spectrometer
LOQ	limit of quantitation
MAP	mean arterial pressure
MAD	mean absolute deviation method
MetS	metabolic syndrome
MS	mass spectra
MPM	microbial phenolic metabolites
OR	odds ratio
SBP	systolic blood pressure
TG	triglycerides
UM	Urinary metabotypes
WC	waist circumference
