# Supplementary material for: Microbial Phenolic Metabolites in Urine Are Inversely Linked to Certain Features of Metabolic Syndrome in Spanish Adolescents
Source: Antioxidants (Basel). 2022 Nov 5;11(11):2191. doi: 10.3390/antiox11112191 (PMC9686597; doi:10.3390/antiox11112191)
Supplement: Supplementary file 1 [file antioxidants-11-02191-s001.zip › antioxidants-1971151-supplementary.pdf]

Supplementary data

Microbial Phenolic Metabolites in Urine are Inversely Linked to Certain Features of Metabolic Syndrome in Spanish Adolescents

Content:

Figure S1. Flow-chart of study participants

Figure S2. Analysis flow-chart

Table S1. Phenolic metabolites identified and quantified in urine by HPLC-LTQ-Orbitrap-HRMS

Table S2. Log-transformed group of MPM and cardiometabolic health parameters

Table S3. Log-transformed group of MPM and MetS and its components

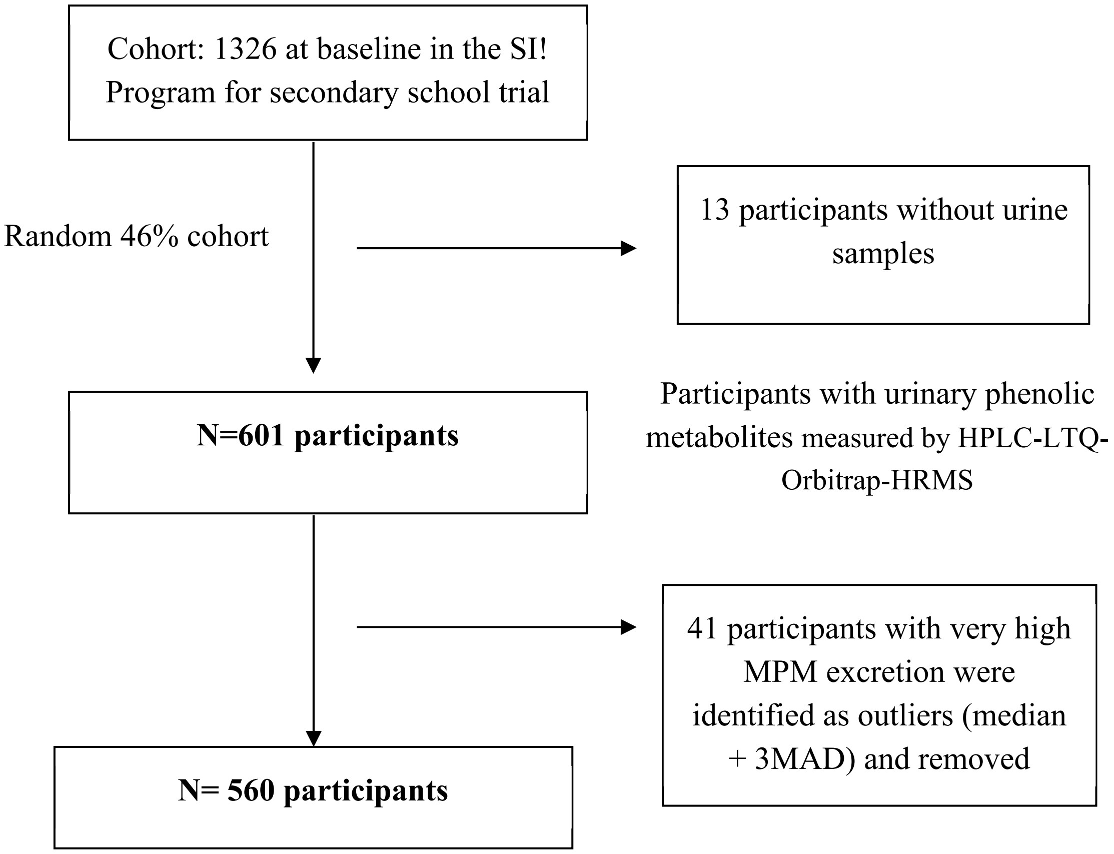

Figure S1. Flow-chart of study participants.

HPLC-LTQ-Orbitrap-HRMS High-performance liquid chromatography coupled to linear trap quadrupole Orbitrap high-resolution mass spectrometer, MAD median absolute deviation, MPM microbial phenolic metabolites.

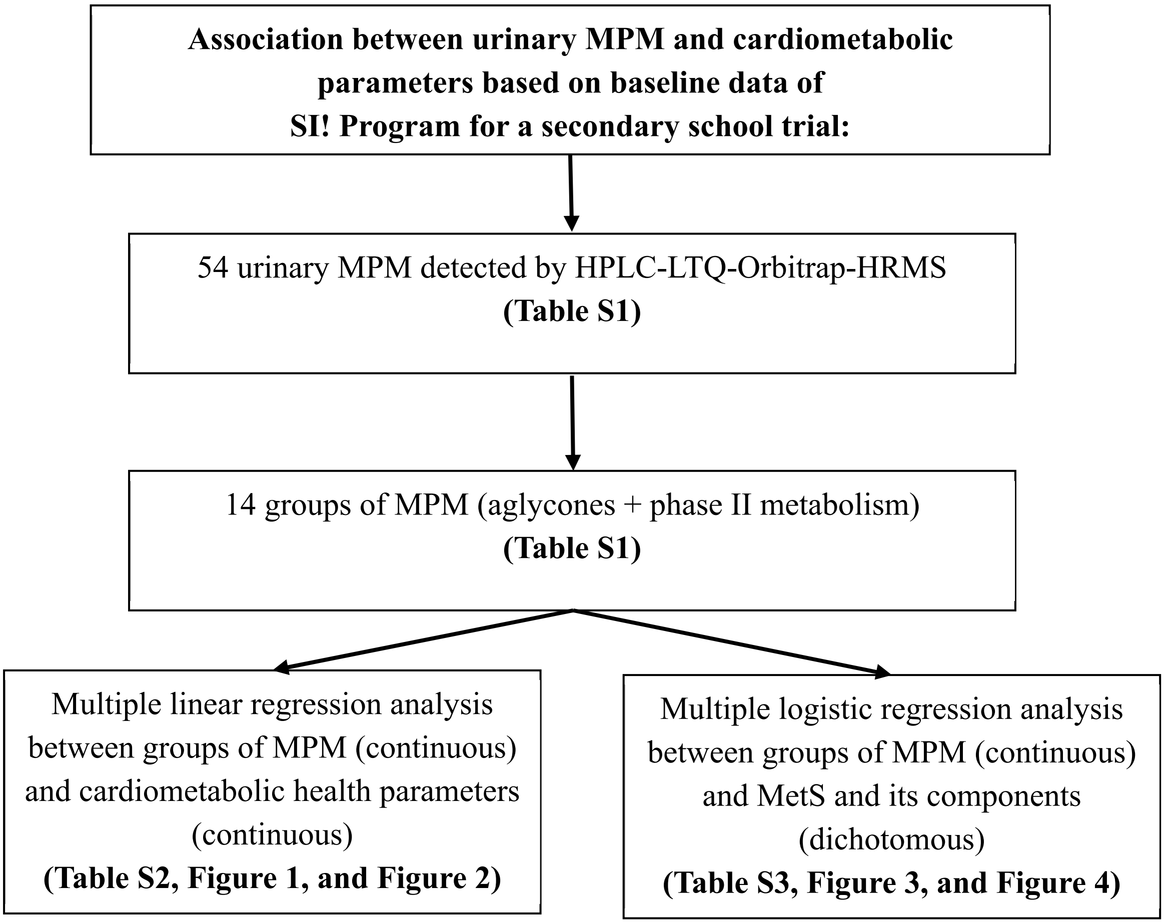

Figure S2. Analysis flow-chart.

HPLC-LTQ-Orbitrap-HRMS High-performance liquid chromatography coupled to linear trap quadrupole Orbitrap high-resolution mass spectrometer, MetS metabolic syndrome, MPM microbial phenolic metabolites.

Table S1. Phenolic metabolites identified and quantified in urine by HPLC-LTQ-Orbitrap-HRMS.

Compound	Neutral Molecular Formula	R_t (min)
**Coumaric acids**		
m-coumaric acid [a]	$C_9H_8O_3$	6.54
o-coumaric acid [a]	$C_9H_8O_3$	7.20
p-coumaric acid [a]	$C_9H_8O_3$	5.96
Coumaric acid glucuronide I (CA)	$C_{15}H_{16}O_9$	4.77
Coumaric acid glucuronide II (CA)	$C_{15}H_{16}O_9$	5.09
Coumaric acid glucuronide III (CA)	$C_{15}H_{16}O_9$	5.82
Coumaric acid sulfate I (CA)	$C_9H_8O_6S$	3.53
Coumaric acid sulfate II (CA)	$C_9H_8O_6S$	4.27
Coumaric acid sulfate III (CA)	$C_9H_8O_6S$	4.74
**Dihydroxyphenylpropionic acid**		
3,4-dihydroxyphenylpropionic acid [a]	$C_9H_{10}O_4$	3.84
Dihydroxyphenylpropionic acid sulfate (3,4-DHPPA)	$C_9H_{10}O_7S$	2.52
**Dihydroresveratrol**		
Dihydroresveratrol [a]	$C_{14}H_{14}O_3$	8.73
Dihydroresveratrol sulfate I (DHRSV)	$C_{14}H_{14}O_6S$	7.07
Dihydroresveratrol sulfate II (DHRSV)	$C_{14}H_{14}O_6S$	7.40
**Enterodiol**		
Enterodiol [a]	$C_{18}H_{22}O_4$	9.17
Enterodiol glucuronide I (ED)	$C_{24}H_{30}O_{10}$	7.48
Enterodiol glucuronide II (ED)	$C_{24}H_{30}O_{10}$	7.61
Enterodiol sulfate (ED)	$C_{18}H_{20}O_7S$	7.50
**Enterolactone**		
Enterolactone [a]	$C_{18}H_{18}O_4$	10.80
Enterolactone glucuronide (EL)	$C_{24}H_{26}O_{10}$	8.77
Enterolactone diglucuronide (EL)	$C_{30}H_{34}O_{16}$	7.24
Enterolactone sulfate (EL)	$C_{18}H_{18}O_7S$	8.57
**Gallic acid**		
Gallic acid [a]	$C_7H_6O_5$	1.69
Gallic acid glucuronide (GA)	$C_{13}H_{14}O_{11}$	1.85
Gallic acid sulfate (GA)	$C_7H_6O_8S$	1.72
**Hydroxybenzoic acid**		
3- hydroxybenzoic acid [a]	$C_7H_6O_3$	4.02
4-hydroxybenzoic acid [a]	$C_7H_6O_3$	3.15
Hydroxybenzoic acid glucuronide I (HBA)	$C_{13}H_{14}O_9$	2.00
Hydroxybenzoic acid glucuronide II (HBA)	$C_{13}H_{14}O_9$	2.50
Hydroxybenzoic acid sulfate (HBA)	$C_7H_6O_6S$	1.89

**Hydroxyphenylacetic acid**		
3-hydroxyphenylacetic acid [a]	$C_8H_8O_3$	4.09
Hydroxyphenylacetic acid glucuronide (3-HPAA)	$C_{14}H_{16}O_9$	4.8
Hydroxyphenylacetic acid sulfate (3-HPAA)	$C_8H_8O_6S$	2.24
**Hydroxytyrosol**		
3-hydroxytyrosol [a]	$C_8H_{10}O_3$	2.11
3′hydroxytyrosol-3′-glucuronide [a]	$C_{14}H_{18}O_9$	2.11
Hydroxytyrosol sulfate (3-HT)	$C_8H_{10}O_6S$	1.82
**Protocatechuic acid**		
Protocatechuic acid [a]	$C_7H_6O_4$	2.25
Protocatechuic acid glucuronide (PCA)	$C_{13}H_{14}O_{10}$	1.67
Protocatechuic acid sulfate I (PCA)	$C_7H_6O_7S$	1.75
Protocatechuic acid sulfate II (PCA)	$C_7H_6O_7S$	1.94
**Syringic acid**		
Syringic acid [a]	$C_9H_{10}O_5$	5.63
Syringic acid glucuronide I (SA)	$C_{15}H_{18}O_{11}$	4.68
Syringic acid glucuronide II (SA)	$C_{15}H_{18}O_{11}$	4.88
Syringic acid sulfate (SA)	$C_9H_{10}O_8S$	4.24
**Urolithin A**		
Urolithin A [a]	$C_{13}H_8O_4$	9.56
Urolithin A glucuronide (Uro A)	$C_{19}H_{16}O_{10}$	6.98
Urolithin A diglucuronide (Uro A)	$C_{25}H_{24}O_{16}$	6.88
Urolithin A sulfate (Uro A)	$C_{13}H_8O_7S$	7.49
**Urolithin B**		
Urolithin B [a]	$C_{13}H_8O_3$	11.12
Urolithin B glucuronide (Uro B)	$C_{19}H_{16}O_9$	8.51
**Vanillic acid**		
Vanillic acid [a]	$C_8H_8O_4$	4.74
Vanillic acid glucuronide I (VA)	$C_{14}H_{16}O_{10}$	2.31
Vanillic acid glucuronide II (VA)	$C_{14}H_{16}O_{10}$	2.76
Vanillic acid sulfate (VA)	$C_8H_8O_7S$	2.03

[a] Commercial standards, Rt retention time, CA coumaric acid, 3,4-DHPPA 3,4-dihydroxyphenylpropionic acid, 3-HPAA 3-hydroxyphenylacetic acid, 3-HBA 3-hydroxybenzoic acid, 3-HT 3-hydroxytyrosol, 3-HT-G 3-Hydroxytyrosol glucuronide, 4-HBA 4-hydroxybenzoic acid, DHRSV Dihydroresveratrol, ED enterodiol, EL enterolactone, GA gallic acid, PCA protocatechuic acid, SA syringic acid, Uro A urolithin A, Uro B urolithin B, VA vanillic acid. When standards were not available, the aglycone was used for quantification (shown in brackets).

Table S2. Log-transformed group of MPM and cardiometabolic health parameters.

	WC z-score			SBP z-score			DBP z-score			TG			HDL-c			BG			MetS score		
	β (95 %CI)	*p*-value	*p*-adj*	β (95% CI)	*p*-value	*p*-adj*	β (95% CI)	*p*-value	*p*-adj*	β (95% CI)	*p*-value	*p*-adj*	β (95% CI)	*p*-value	*p*-adj*	β (95% CI)	*p*-value	*p*-adj*	β (95% CI)	*p*-value	*p*-adj*
**Coumaric acids**																					
Model 1	0.01 (-0.02;0.04)	0.523	0.666	0.00 (-0.04;0.04)	0.976	0.976	-0.01 (-0.04;0.03)	0.705	0.868	1.75 (0.18;3.33)	**0.030**	0.330	-0.2 (-0.73;0.35)	0.484	0.847	0.27 (-0.19;0.72)	0.254	0.889	0.08 (-0.03;0.18)	0.144	0.271
Model 2	-0.01 (-0.04;0.03)	0.646	0.822	-0.02 (-0.06;0.02)	0.343	0.437	-0.03 (-0.06;0.01)	0.142	0.387	1.54 (-0.23;3.3)	0.088	0.411	0.02 (-0.58;0.61)	0.956	0.956	0.43 (-0.1;0.94)	0.108	0.756	0.04 (-0.08;0.15)	0.513	0.798
**Dihydroxyphenylpropionic acid**																					
Model 1	-0.01 (-0.02;0.01)	0.608	0.709	-0.01 (-0.03;0.01)	0.180	0.360	-0.01 (-0.02;0.01)	0.444	0.777	0.09 (-0.58;0.75)	0.799	0.974	0.20 (-0.03;0.43)	0.086	0.569	0.02 (-0.18;0.21)	0.853	0.919	-0.02 (-0.07;0.03)	0.394	0.549
Model 2	-0.01 (-0.02;0.01)	0.735	0.858	-0.02 (-0.03;0.00)	**0.048**	0.231	-0.01 (-0.02;0.01)	0.249	0.387	0.11 (-0.61;0.83)	0.766	0.972	0.12 (-0.13;0.36)	0.351	0.956	0.02 (-0.2;0.24)	0.870	0.948	-0.02 (-0.06;0.04)	0.572	0.801
**Dihydroresveratrol**																					
Model 1	-0.01 (-0.02;0.01)	0.475	0.666	-0.02 (-0.04;0.01)	0.093	0.301	-0.01 (-0.02;0.01)	0.313	0.626	0.07 (-0.75;0.89)	0.870	0.974	0.22 (-0.06;0.5)	0.122	0.569	-0.09 (-0.32;0.15)	0.468	0.919	-0.04 (-0.09;0.02)	0.155	0.271
Model 2	-0.01 (-0.03;0.01)	0.505	0.786	-0.02 (-0.04;0.00)	**0.048**	0.231	-0.01 (-0.03;0.01)	0.204	0.387	0.24 (-0.68;1.15)	0.612	0.972	0.08 (-0.23;0.38)	0.623	0.956	-0.07 (-0.34;0.2)	0.611	0.948	-0.03 (-0.09;0.03)	0.345	0.634
**Enterodiol**																					
Model 1	-0.01 (-0.03;0.02)	0.441	0.666	-0.02 (-0.05;0.01)	0.126	0.301	-0.02 (-0.04;0.01)	0.101	0.445	0.51 (-0.67;1.67)	0.399	0.931	-0.05 (-0.45;0.35)	0.803	0.950	-0.07 (-0.4;0.27)	0.704	0.919	-0.03 (-0.11;0.05)	0.431	0.549
Model 2	0.00 (-0.03;0.03)	0.981	0.981	-0.02 (-0.04;0.02)	0.275	0.385	-0.02 (-0.04;0.01)	0.219	0.387	0.42 (-0.85;1.69)	0.516	0.972	-0.07 (-0.49;0.35)	0.741	0.956	-0.03 (-0.4;0.34)	0.880	0.948	-0.01 (-0.09;0.08)	0.851	0.866
**Enterolactone**																					
Model 1	-0.04 (-0.08;0.01)	0.115	0.350	-0.05 (-0.1;0.01)	0.058	0.301	-0.03 (-0.07;0.02)	0.159	0.445	-1.89 (-4.19;0.41)	0.107	0.330	0.42 (-0.39;1.23)	0.306	0.774	-0.15 (-0.82;0.54)	0.682	0.919	-0.17 (-0.32;-0.02)	**0.033**	0.231
Model 2	-0.03 (-0.08;0.02)	0.178	0.498	-0.05 (-0.1;0.01)	0.053	0.231	-0.04 (-0.08;0.01)	0.129	0.387	-1.22 (-3.66;1.22)	0.326	0.913	0.03 (-0.81;0.87)	0.945	0.956	-0.01 (-0.74;0.73)	0.988	0.988	-0.12 (-0.28;0.04)	0.129	0.602
**Gallic acid**																					
Model 1	-0.01 (-0.03;0.01)	0.239	0.478	-0.01 (-0.03;0.02)	0.498	0.697	-0.01 (-0.02;0.02)	0.926	0.955	-0.67 (-1.42;0.09)	0.082	0.330	0.08 (-0.21;0.37)	0.581	0.904	-0.08 (-0.32;0.17)	0.558	0.919	-0.05 (-0.1;0.01)	0.100	0.271
Model 2	-0.02 (-0.03;-0.01)	**0.041**	0.287	-0.02 (-0.03;0.01)	0.189	0.378	-0.01 (-0.02;0.01)	0.542	0.747	-0.8 (-1.59;-0.01)	**0.048**	0.411	0.19 (-0.11;0.49)	0.200	0.933	-0.14 (-0.4;0.12)	0.291	0.948	-0.07 (-0.13;-0.02)	**0.009**	0.063
**Hydroxybenzoic acid**																					

Model 1	0.04 (-0.01;0.09)	0.097	0.350	0.01 (-0.05;0.06)	0.796	0.976	0.01 (-0.04;0.05)	0.744	0.868	0.16 (-2.33;2.64)	0.904	0.974	-0.03 (-0.91;0.85)	0.950	0.950	0.45 (-0.3;1.2)	0.235	0.889	0.10 (-0.07;0.27)	0.232	0.361
Model 2	0.04 (-0.02;0.09)	0.137	0.480	-0.01 (-0.06;0.06)	0.931	0.983	0.01 (-0.05;0.05)	0.942	0.949	0.14 (-2.57;2.84)	0.923	0.972	-0.32 (-1.26;0.62)	0.505	0.956	0.4 (-0.44;1.23)	0.348	0.948	0.10 (-0.08;0.27)	0.284	0.634
**Hydroxyphenylacetic acid**																					
Model 1	0.01 (-0.02;0.02)	0.860	0.926	0.02 (-0.01;0.03)	0.066	0.301	-0.01 (-0.02;0.01)	0.696	0.868	-0.09 (-0.82;0.65)	0.818	0.974	0.03 (-0.23;0.29)	0.818	0.950	0.04 (-0.18;0.26)	0.736	0.919	0.00 (-0.05;0.05)	0.986	0.986
Model 2	0.01 (-0.01;0.02)	0.635	0.822	0.02 (-0.01;0.04)	0.066	0.231	-0.01 (-0.02;0.01)	0.587	0.747	-0.08 (-0.89;0.73)	0.847	0.972	0.03 (-0.25;0.3)	0.864	0.956	0.06 (-0.19;0.3)	0.653	0.948	0.01 (-0.05;0.06)	0.855	0.866
**Hydroxytyrosol**																					
Model 1	0.01 (-0.01;0.02)	0.517	0.666	-0.01 (-0.02;0.02)	0.851	0.976	-0.01 (-0.02;0.01)	0.295	0.626	-0.12 (-0.9;0.67)	0.772	0.974	-0.06 (-0.33;0.22)	0.698	0.950	-0.06 (-0.29;0.18)	0.643	0.919	-0.01 (-0.06;0.05)	0.720	0.775
Model 2	0.01 (-0.01;0.03)	0.347	0.607	-0.01 (-0.03;0.02)	0.445	0.519	-0.01 (-0.03;0.01)	0.247	0.387	-0.03 (-0.89;0.84)	0.954	0.972	-0.26 (-0.55;0.04)	0.090	0.630	-0.04 (-0.3;0.23)	0.789	0.948	0.01 (-0.05;0.07)	0.751	0.866
**Protocatechuic acid**																					
Model 1	-0.03 (-0.06;0.01)	0.125	0.350	-0.03 (-0.07;0.01)	0.129	0.301	-0.03 (-0.05;0.01)	0.127	0.445	0.01 (-1.6;1.6)	0.998	0.998	0.61 (0.03;1.19)	**0.040**	0.560	0.06 (-0.44;0.55)	0.820	0.919	-0.1 (-0.21;0.02)	0.081	0.271
Model 2	-0.02 (-0.05;0.02)	0.276	0.552	-0.02 (-0.06;0.02)	0.261	0.385	-0.02 (-0.05;0.02)	0.247	0.387	-0.32 (-1.98;1.34)	0.705	0.972	0.52 (-0.07;1.11)	0.083	0.630	0.06 (-0.47;0.58)	0.840	0.948	-0.08 (-0.19;0.04)	0.175	0.613
**Syringic acid**																					
Model 1	0.01 (-0.01;0.03)	0.194	0.453	0.00 (-0.02;0.02)	0.965	0.976	0.00 (-0.02;0.02)	0.955	0.955	0.69 (-0.18;1.54)	0.118	0.330	-0.14 (-0.44;0.17)	0.387	0.774	0.21 (-0.05;0.45)	0.114	0.798	0.05 (-0.02;0.1)	0.148	0.271
Model 2	0.02 (-0.01;0.03)	0.120	0.480	0.00 (-0.02;0.02)	0.983	0.983	-0.01 (-0.02;0.02)	0.719	0.839	0.56 (-0.38;1.49)	0.245	0.858	-0.1 (-0.41;0.23)	0.572	0.956	0.15 (-0.14;0.43)	0.310	0.948	0.04 (-0.03;0.1)	0.246	0.634
**Urolithin A**																					
Model 1	-0.01 (-0.02;0.01)	0.090	0.350	-0.01 (-0.02;0.01)	0.112	0.301	-0.01 (-0.02;-0.01)	**0.011**	0.154	-0.1 (-0.57;0.37)	0.686	0.974	0.01 (-0.16;0.18)	0.906	0.950	-0.01 (-0.15;0.14)	0.986	0.986	-0.03 (-0.06;0.01)	0.154	0.271
Model 2	-0.01 (-0.02;0.01)	0.218	0.509	-0.01 (-0.02;0.01)	0.130	0.303	-0.02 (-0.03;-0.01)	**0.003**	**0.042**	-0.02 (-0.52;0.49)	0.957	0.972	-0.02 (-0.2;0.16)	0.839	0.956	0.03 (-0.13;0.19)	0.706	0.948	-0.02 (-0.05;0.02)	0.362	0.634
**Urolithin B**																					
Model 1	-0.02 (-0.03;-0.01)	**0.003**	**0.042**	-0.01 (-0.03;0.01)	0.229	0.401	-0.01 (-0.03;0.01)	0.104	0.445	-0.55 (-1.17;0.07)	0.081	0.330	0.11 (-0.14;0.36)	0.381	0.774	-0.18 (-0.38;0.03)	0.094	0.798	-0.07 (-0.11;-0.02)	**0.005**	0.070
Model 2	-0.03 (-0.04;-0.01)	**<0.001**	**0.006**	-0.02 (-0.03;0.01)	0.102	0.286	-0.02 (-0.03;-0.003)	**0.013**	0.091	-0.63 (-1.29;0.03)	0.059	0.411	0.11 (-0.16;0.36)	0.425	0.956	-0.25 (-0.47;-0.04)	**0.024**	0.336	-0.08 (-0.12;-0.04)	**0.001**	**0.014**
**Vanillic acid**																					
Model 1	0.00 (-0.04;0.04)	0.986	0.986	0.02 (-0.03;0.07)	0.423	0.658	-0.01 (-0.05;0.03)	0.604	0.868	-0.2 (-2.42;2.04)	0.864	0.974	0.42 (-0.36;1.19)	0.296	0.774	0.17 (-0.49;0.82)	0.619	0.919	-0.04 (-0.19;0.11)	0.619	0.722
Model 2	0.01 (-0.04;0.05)	0.823	0.886	0.03 (-0.02;0.08)	0.262	0.385	-0.01 (-0.05;0.04)	0.949	0.949	0.05 (-2.3;2.38)	0.972	0.972	0.33 (-0.47;1.12)	0.422	0.956	0.22 (-0.48;0.92)	0.537	0.948	-0.02 (-0.17;0.14)	0.866	0.866

β estimated beta, BG blood glucose CI confidence interval, DBP diastolic blood pressure, HDL-c high-density lipoprotein-cholesterol, MetS metabolic syndrome, MPM microbial phenolic metabolites, SBP systolic blood pressure, TG triglycerides, WC waist circumference. Mixed-effects linear regression models between log-transformed group of MPM (aglycone plus phase II metabolites) and each cardiometabolic health parameter. Fixed effect included two models: model 1 included sex (female/male) and age (continuous, years); Model 2 included model 1 plus Tanner maturation stage (score from I to V), physical activity (≥ 60min / <60min moderate-to-vigorous physical activity), household income (low/medium/high), and energy intake (continuous, Kcal/day). Municipality and schools were considered as random effects. * *p*-adjusted for multiple-testing using the Benjamin-Hochberg procedure considering a false discovery rate < 0.05 as significant. P values < 0.05 were considered significant.

Table S3. Log-transformed group of MPM and MetS and its components.

	WC ≥ 90th			TG ≥ 150 mg/dL			HDL-c ≤ 40 mg/dL			BG ≥ 110 mg/dL			MetS		
	**OR (95 %CI)**	***p*-value**	***p*-adj***	**OR (95 %CI)**	***p*-value**	***p*-adj***	**OR (95 %CI)**	***p*-value**	***p*-adj***	**OR (95 %CI)**	***p*-value**	***p*-adj***	**OR (95 %CI)**	***p*-value**	***p*-adj***
**Coumaric acids**															
Model 1	1.06 (0.96;1.17)	0.312	0.485	1.08 (0.90;1.29)	0.399	0.868	1.02 (0.85;1.23)	0.821	0.915	1.02 (0.94;1.11)	0.637	0.811	0.96 (0.79;1.18)	0.714	0.859
Model 2	1.04 (0.93;1.16)	0.580	0.756	1.06 (0.86;1.30)	0.595	0.944	0.96 (0.81;1.15)	0.687	0.802	1.04 (0.94;1.16)	0.430	0.754	0.93 (0.74;1.18)	0.562	0.715
**Dihydroxyphenylpropionic acid**															
Model 1	0.99 (0.96;1.03)	0.526	0.669	1.00 (0.95;1.06)	0.991	0.991	0.94 (0.89;1.00)	0.050	0.294	0.99 (0.96;1.03)	0.705	0.823	0.98 (0.90;1.06)	0.603	0.859
Model 2	1.00 (0.96;1.04)	0.648	0.756	1.00 (0.94;1.07)	0.944	0.944	0.93 (0.87;0.99)	**0.048**	0.437	1.00 (0.96;1.04)	0.922	0.953	1.01 (0.90;1.13)	0.897	0.897
**Dihydroresveratrol**															
Model 1	0.99 (0.95;1.03)	0.395	0.553	1.01 (0.94;1.08)	0.854	0.991	0.97 (0.90;1.05)	0.449	0.698	0.98 (0.94;1.02)	0.333	0.666	1.02 (0.91;1.14)	0.736	0.859
Model 2	0.99 (0.94;1.04)	0.455	0.708	1.01 (0.93;1.09)	0.894	0.944	0.99 (0.90;1.09)	0.876	0.943	0.98 (0.94;1.03)	0.431	0.754	1.09 (0.90;1.32)	0.356	0.623
**Enterodiol**															
Model 1	0.97 (0.91;1.02)	0.180	0.315	1.02 (0.92;1.12)	0.753	0.991	1.04 (0.90;1.20)	0.580	0.812	0.97 (0.92;1.03)	0.308	0.666	1.03 (0.87;1.23)	0.707	0.859
Model 2	0.99 (0.92;1.06)	0.610	0.756	1.01 (0.90;1.13)	0.871	0.944	1.04 (0.89;1.21)	0.653	0.802	0.98 (0.92;1.04)	0.582	0.815	1.02 (0.85;1.23)	0.798	0.859
**Enterolactone**															
Model 1	0.90 (0.79;1.02)	0.084	0.201	0.83 (0.71;0.98)	0.026	0.364	0.82 (0.68;1.00)	0.052	0.294	0.96 (0.85;1.09)	0.514	0.806	0.78 (0.59;1.02)	0.064	0.299
Model 2	0.90 (0.78;1.05)	0.162	0.397	0.86 (0.72;1.04)	0.116	0.717	0.85 (0.68;1.06)	0.156	0.437	1.01 (0.88;1.16)	0.859	0.953	0.80 (0.59;1.09)	0.165	0.483
**Gallic acid**															
Model 1	0.96 (0.93;1)	**0.048**	0.201	0.99 (0.93;1.04)	0.618	0.991	1.00 (0.91;1.09)	0.992	0.992	0.97 (0.93;1.01)	0.200	0.666	0.88 (0.81;0.96)	**0.003**	**0.042**
Model 2	0.93 (0.89;0.98)	**0.002**	**0.021**	0.97 (0.91;1.03)	0.345	0.805	0.97 (0.88;1.07)	0.536	0.802	0.96 (0.92;1.00)	0.066	0.308	0.85 (0.77;0.93)	**0.001**	**0.014**
**Hydroxybenzoic acid**															

Model 1	1.14 (0.99;1.32)	0.086	0.201	0.99 (0.82;1.21)	0.958	0.991	0.98 (0.77;1.24)	0.850	0.915	0.99 (0.86;1.13)	0.852	0.852	0.97 (0.67;1.40)	0.851	0.916
Model 2	1.17 (0.99;1.39)	0.073	0.256	0.98 (0.78;1.24)	0.866	0.944	0.94 (0.71;1.25)	0.657	0.802	1.00 (0.86;1.17)	0.953	0.953	1.06 (0.69;1.64)	0.789	0.859
**Hydroxyphenylacetic acid**															
Model 1	1.00 (0.96;1.04)	0.713	0.769	0.98 (0.93;1.03)	0.434	0.868	1.05 (0.95;1.16)	0.337	0.674	1.00 (0.97;1.04)	0.819	0.852	0.97 (0.90;1.06)	0.531	0.859
Model 2	1.01 (0.97;1.06)	0.755	0.772	0.97 (0.92;1.02)	0.256	0.717	1.04 (0.93;1.16)	0.492	0.802	1.01 (0.97;1.06)	0.527	0.815	0.97 (0.89;1.06)	0.499	0.699
**Hydroxytyrosol**															
Model 1	1.00 (0.96;1.05)	0.998	0.998	0.99 (0.93;1.06)	0.829	0.991	0.96 (0.89;1.03)	0.245	0.656	0.99 (0.95;1.03)	0.576	0.806	0.94 (0.86;1.02)	0.115	0.369
Model 2	1.01 (0.96;1.06)	0.772	0.772	0.99 (0.92;1.06)	0.789	0.944	0.94 (0.86;1.02)	0.134	0.437	1.00 (0.95;1.04)	0.886	0.953	0.91 (0.83;0.99)	**0.027**	0.189
**Protocatechuic acid**															
Model 1	0.93 (0.85;1.02)	0.102	0.204	1.11 (0.98;1.27)	0.102	0.597	0.95 (0.82;1.09)	0.448	0.698	0.93 (0.85;1.01)	0.101	0.471	1.00 (0.79;1.27)	0.982	0.982
Model 2	0.94 (0.85;1.04)	0.170	0.397	1.13 (0.98;1.31)	0.099	0.717	0.95 (0.82;1.10)	0.483	0.802	0.94 (0.85;1.03)	0.182	0.425	0.90 (0.73;1.11)	0.317	0.623
**Syringic acid**															
Model 1	1.06 (1;1.12)	0.079	0.201	1.10 (0.97;1.25)	0.128	0.597	1.08 (0.94;1.26)	0.281	0.656	1.03 (0.98;1.08)	0.252	0.666	1.17 (0.85;1.61)	0.338	0.676
Model 2	1.11 (1.02;1.21)	**0.019**	0.089	1.07 (0.95;1.21)	0.254	0.717	1.06 (0.92;1.21)	0.427	0.802	1.04 (0.98;1.09)	0.178	0.425	1.13 (0.83;1.52)	0.439	0.683
**Urolithin A**															
Model 1	0.98 (0.96;1.01)	0.071	0.201	1.00 (0.96;1.03)	0.885	0.991	0.95 (0.90;1.01)	0.104	0.364	0.97 (0.94;0.99)	**0.020**	0.140	0.95 (0.89;1.02)	0.152	0.369
Model 2	0.99 (0.96;1.02)	0.271	0.542	1.00 (0.96;1.05)	0.873	0.944	0.95 (0.89;1.01)	0.103	0.437	0.97 (0.94;0.99)	**0.024**	0.168	0.95 (0.88;1.03)	0.206	0.483
**Urolithin B**															
Model 1	0.95 (0.92;0.99)	**0.010**	0.140	0.97 (0.92;1.02)	0.248	0.694	0.92 (0.84;1.00)	0.063	0.294	0.93 (0.90;0.97)	**<0.001**	**0.006**	0.88 (0.77;1.00)	0.057	0.299
Model 2	0.94 (0.89;0.98)	**0.003**	**0.021**	0.98 (0.92;1.03)	0.436	0.872	0.93 (0.83;1.03)	0.152	0.437	0.92 (0.88;0.96)	**<0.001**	**0.001**	0.85 (0.73;0.99)	**0.048**	0.224
**Vanillic acid**															
Model 1	1.03 (0.91;1.17)	0.714	0.769	1.12 (0.92;1.37)	0.248	0.694	1.06 (0.82;1.36)	0.679	0.864	1.04 (0.92;1.17)	0.530	0.806	1.30 (0.90;1.88)	0.158	0.369

Model 2	1.07 (0.92;1.24)	0.436	0.708	1.15 (0.92;1.43)	0.232	0.717	1.00 (0.76;1.32)	0.998	0.998	1.12 (0.96;1.29)	0.143	0.425	1.30 (0.87;1.94)	0.207	0.483

BG blood glucose, CI confidence interval, HDL-c high-density lipoprotein-cholesterol, MetS metabolic syndrome, MPM microbial phenolic metabolites, TG triglycerides, WC waist circumference. Mixed-effects logistic regression models between log-transformed group of MPM (aglycone plus phase II metabolites) and MetS and its components. Fixed effect included two models: model 1 included sex (female/male) and age (continuous, years); Model 2 included model 1 plus Tanner maturation stage (score from I to V), physical activity (≥ 60min / <60min moderate-to-vigorous physical activity), household income (low/medium/high), and energy intake (continuous, Kcal/day). Municipality and schools were considered random effects. High blood pressure was not considered in the statistical analysis as only 2 participants had this condition (systolic blood pressure ≥130 mm Hg or diastolic blood pressure ≥ 85 mm Hg), and therefore the analysis did not converge. * p-adjusted for multiple-testing using the Benjamin-Hochberg procedure considering a false discovery rate < 0.05 as significant. P values < 0.05 were considered significant.
